# Lineage Tracing of Cardiac Explant Derived Cells

**DOI:** 10.1371/journal.pone.0001929

**Published:** 2008-04-16

**Authors:** Lincoln T. Shenje, Loren J. Field, Catrin A. Pritchard, Christopher J. Guerin, Michael Rubart, Mark H. Soonpaa, Keng-Leong Ang, Manuel Galiñanes

**Affiliations:** 1 Cardiac Surgery Unit, Department of Cardiovascular Sciences, University of Leicester, Leicester, United Kingdom; 2 Herman B Wells Center for Pediatric Research, Division of Pediatric Cardiology and Krannert Institute of Cardiology, Indiana University School of Medicine, Indianapolis, Indiana, United States of America; 3 Department of Biochemistry, University of Leicester, Leicester, United Kingdom; 4 MRC Toxicology Unit, University of Leicester, Leicester, United Kingdom; Fred Hutchinson Cancer Research Center, United States of America

## Abstract

**Aims:**

Cultured cardiac explants produce a heterogeneous population of cells including a distinctive population of refractile cells described here as small round cardiac explant derived cells (EDCs). The aim of this study was to explore the source, morphology and cardiogenic potential of EDCs.

**Methods:**

Transgenic MLC2v-Cre/ZEG, and actin-eGFP mice were used for lineage-tracing of EDCs *in vitro* and *in vivo*. C57B16 mice were used as cell transplant recipients of EDCs from transgenic hearts, as well as for the general characterisation of EDCs. The activation of cardiac-specific markers were analysed by: immunohistochemistry with bright field and immunofluorescent microscopy, electron microscopy, PCR and RT-PCR. Functional engraftment of transplanted cells was further investigated with calcium transient studies.

**Results:**

Production of EDCs was highly dependent on the retention of blood-derived cells or factors in the cultured explants. These cells shared some characteristics of cardiac myocytes *in vitro* and survived engraftment in the adult heart *in vivo*. However, EDCs failed to differentiate into functional cardiac myocytes *in vivo* as demonstrated by the absence of stimulation-evoked intracellular calcium transients following transplantation into the peri-infarct zone.

**Conclusions:**

This study highlights that positive identification based upon one parameter alone such as morphology or immunofluorescene is not adequate to identify the source, fate and function of adult cardiac explant derived cells.

## Introduction

The identification and isolation of adult cardiac stem cells offers the possibility of developing new treatment strategies for heart disease. It has recently been reported that cells derived from cultured adult human and murine explants have cardiogenic potential[1]. Understanding the biological characteristics of these cardiac explant derived cells would be important in order to gain a better insight of their role in maintaining cardiac function and their therapeutic potential as cardiac progenitors for myocardial repair.

Previous studies have reported that cardiac explant derived cells express markers of stem cells[1], [2]. They showed that after production of a layer of fibroblast-like cells, cultured adult mouse cardiac explants produced cardiogenic small, round “phase bright” cells after 2 to 3 weeks in culture. These results were replicated with biopsies of cultured human cardiac explants[3]. The cells were clonogenic and could be expanded *in vitro* to form cardiospheres, providing the possibility of scalable production for myocardial repair for clinical trials. Following transplantation in experimental models of myocardial infarction, the explant-derived cells differentiated into cardiac myocytes accompanied by an improvement in cardiac function[1], [3]. Additional evidence also shows that stem-like cells derived from cultured cardiac explants improved vascularisation of the injured myocardium[2].

To expand upon these interesting results, we sought to define the source, morphology and cardiogenic potential of a highly refractile population of small, round cardiac explant derived cells, termed here as EDCs, and using different lineage-tracing techniques *in vitro* and *in vivo*.

## Results

### Characteristics of Cultured Cardiac Explants in vitro

**Figure 1:**
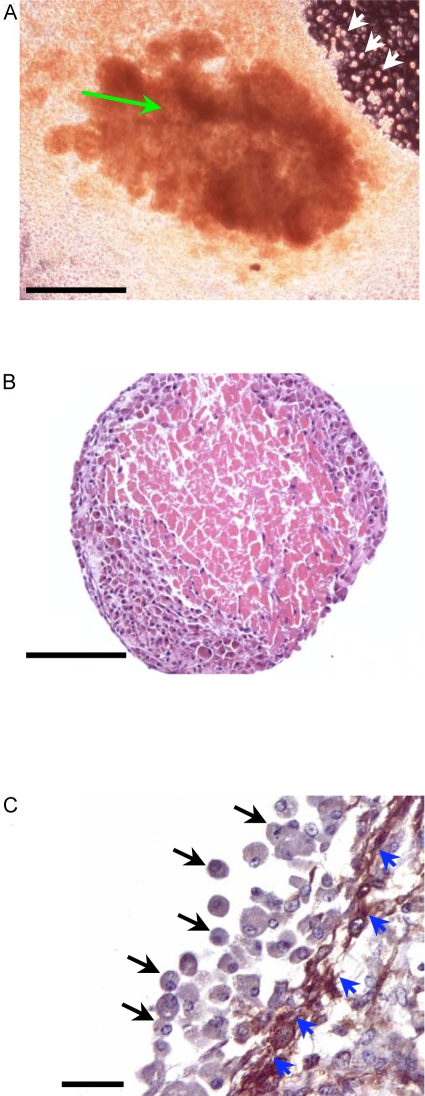
Cultured cardiac explants. (a) Phase contrast image of numerous highly refractile EDCs (white arrows) from a mouse ventricular explant (green arrows) in culture for 3 weeks (scale bar, 100 microns). (b) Hematoxylin and eosin staining of a mouse ventricular explant in culture for 3 weeks showing that the explant has an eosinophilic core of anuclear myocyte remnants and a new region of basophilic cells in the periphery (scale bar, 100 microns). (c) Explants were stained for the presence of smooth muscle actin and counterstained with hematoxylin. EDCs, which do not express smooth muscle actin (black arrows) bud through an epithelial-like layer of fibroblast-like cells (blue arrowheads) that express smooth muscle actin (DAB brown) to reach the surface of the explant (scale bar, 20 microns).

Mouse cardiac muscle obtained from the ventricles of 8–12 week old C57Bl6 mice were diced into small explants and cultured. After a week in culture, the explants became adherent to the culture substrate, the surface becoming smooth and lined by a fibroblast-like layer of cells. From the second week onwards, proliferating fibroblast-like cells migrated radially away from the explants. During the same period, a highly refractile population of small round EDCs, began to bud vigorously through the smooth surface of the explants, forming colonies of EDCs interspersed by fibroblast-like cells. Figure 1a shows the appearance of a typical explant after 3 weeks in culture. Histochemical analyses revealed the presence of eosinophilic anuclear remnants of cardiomyocytes in the centre of the explants (Figure 1b). The fibroblast-like cells lining the surface of the cultured explant were positive for smooth muscle actin (Figure 1c, blue arrows). Only four percent of the EDCs incorporated BrdU after 6 hrs of labelling (data not shown). The formation of EDCs from the explants could be inhibited by cytarabinoside (a cell cycle inhibitor), but resumed when the cytarabinoside was withdrawn.

**Figure 2:**
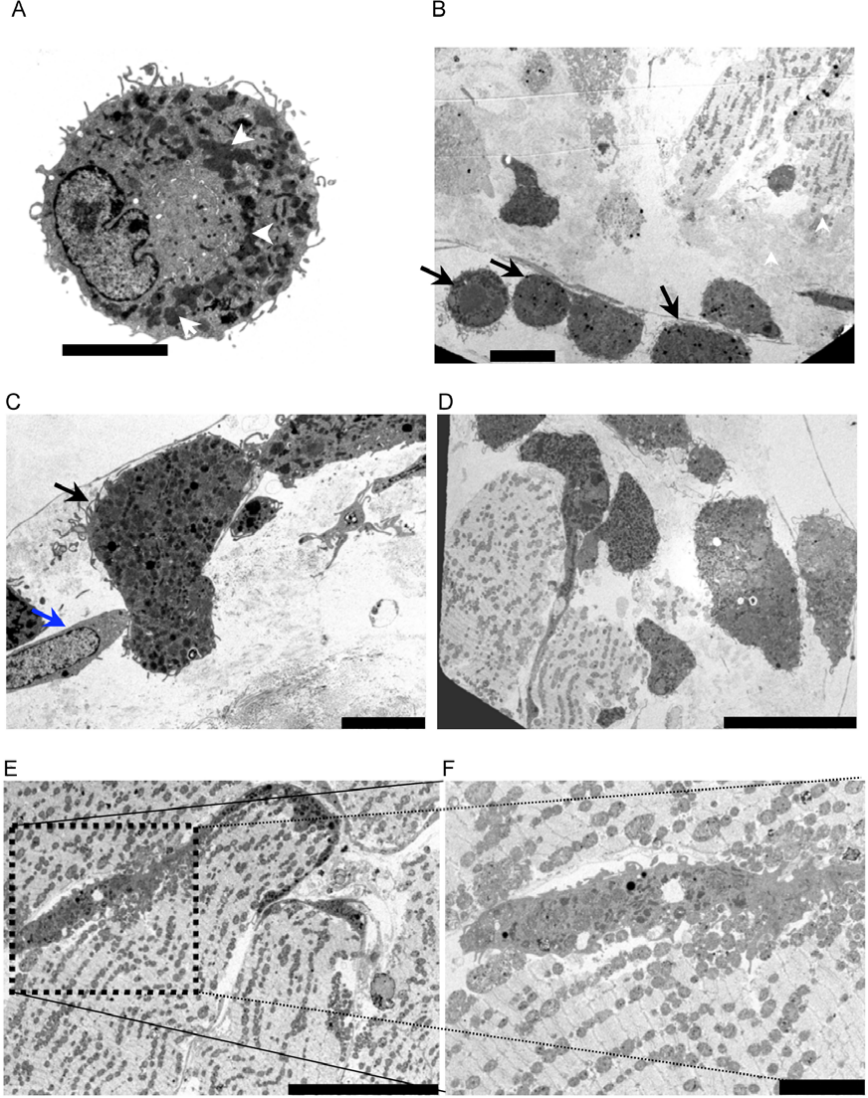
Ultrastructural characteristics of EDCs. (a) EDCs have numerous electron dense cytoplasmic structures (white arrowheads) and pseudopodia (scale bar, 2 microns). (b) View at the surface of a cardiac explant reveals numerous EDCs (black arrows) on the surface and immediately within the explant (scale bar, 5 microns). (c) An EDC (black arrow) near an epithelial-like cell (blue arrow) adjacent to the surface of a cultured cardiac explant after 3 weeks in culture (scale bar 5 microns). (d) Cells with ultrastructural attributes identical to EDCs are present deep within the cultured explants (scale bar, 20 microns). (e) EDC-like cells within the explant interstitium exhibit a large number of dense intercellular inclusions and presumptive lysosomes (scale bar, 20 microns). (f) is the insert from figure 2e (scale bar, 5 microns).

Transmission Electron Microscopy (EM) was employed to further characterise the EDCs. EDCs exhibited multiple electron dense sub-cellular structures in the cytoplasm and numerous fine pseudopodia (Fig 2a). Cells with identical ultrastructural attributes were observed on the surface as well as just within the explant tissue (Figure 2b), and frequently were found to be closely opposed to fibroblast at the edge of the explants (Figure 2c). Similar cells were also present within the core of the culture explants (Figure 2d). EDC-like cells within the explant interstitium exhibit a large number of dense intercellular inclusions (Figure 2e–f).

**Figure 3:**
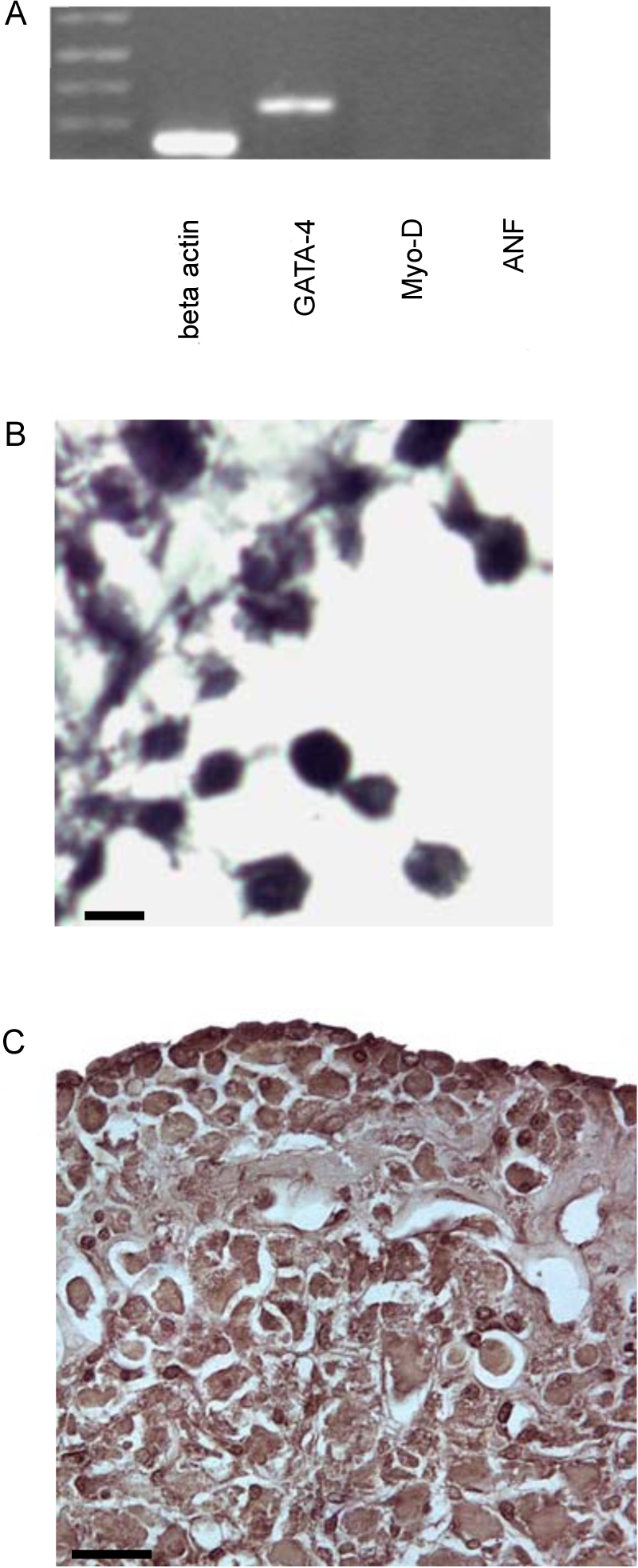
Expression profile of EDCs. (a) RT PCR analysis of EDCs gene expression: Transcripts encoding GATA-4 were detected by no MyoD and ANF transcripts. (b and c) Vimentin and α-sarcomeric actinin immune reactivity, respectively, in cultured EDCs (b-scale bar, 5 microns; c -scale bar, 20 microns).

**Figure 4:**
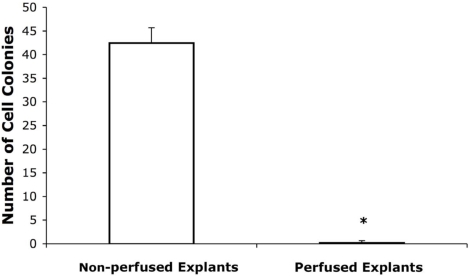
Production of EDCs in the cultured cardiac explants is dependent on the presence of retained circulation elements. Colonies of EDCs were quantified as a percentage of the total of explants per culture dish (one dish per heart, n = 4 hearts/group). *: p<0.05 vs. the non-perfused explant group.

RT-PCR analyses were performed to determine if EDCs expressed cardiac-specific markers. Transcripts encoding GATA4 were detected, but not MyoD, nor ANF transcripts (Figure 3a). The transcriptional factor for NKx2.5 was also undetected (data not shown). Expression of other markers was examined by immune histochemistry. EDCs were stained positive for the mesenchymal marker vimentin (Figure 3b) and α-sarcomeric actinin (Figure 3c), but were negative for the endothelial cell marker von-Willebrand factor and the pericyte marker NG2. Furthermore, they did not express the stem cell markers stem cell antigen (Sca-1) or c-kit (see figure S1). To determine if the EDCs were present in the myocardial interstitium, blood was flushed out of the hearts by retrograde perfusion on a Langendorff apparatus for approximately three minutes at room temperature. Migrating EDCs were observed in only one of 498 explants tested from the perfused hearts (n = 4 mice, see Figure 4), suggesting that EDCs might be blood borne. The observed attributes of the EDCs (i.e., delayed appearance of a highly refractile population of small cells following fibroblast outgrowth, and partial activation of a cardiomyogenic program) were consistent with other studies describing cardiomyogeic stem cells from explanted heart tissue[1]–[3].

### Cardiomyogenic Lineage Tracing in EDCs using a Transgenic Reporter Model

**Figure 5:**
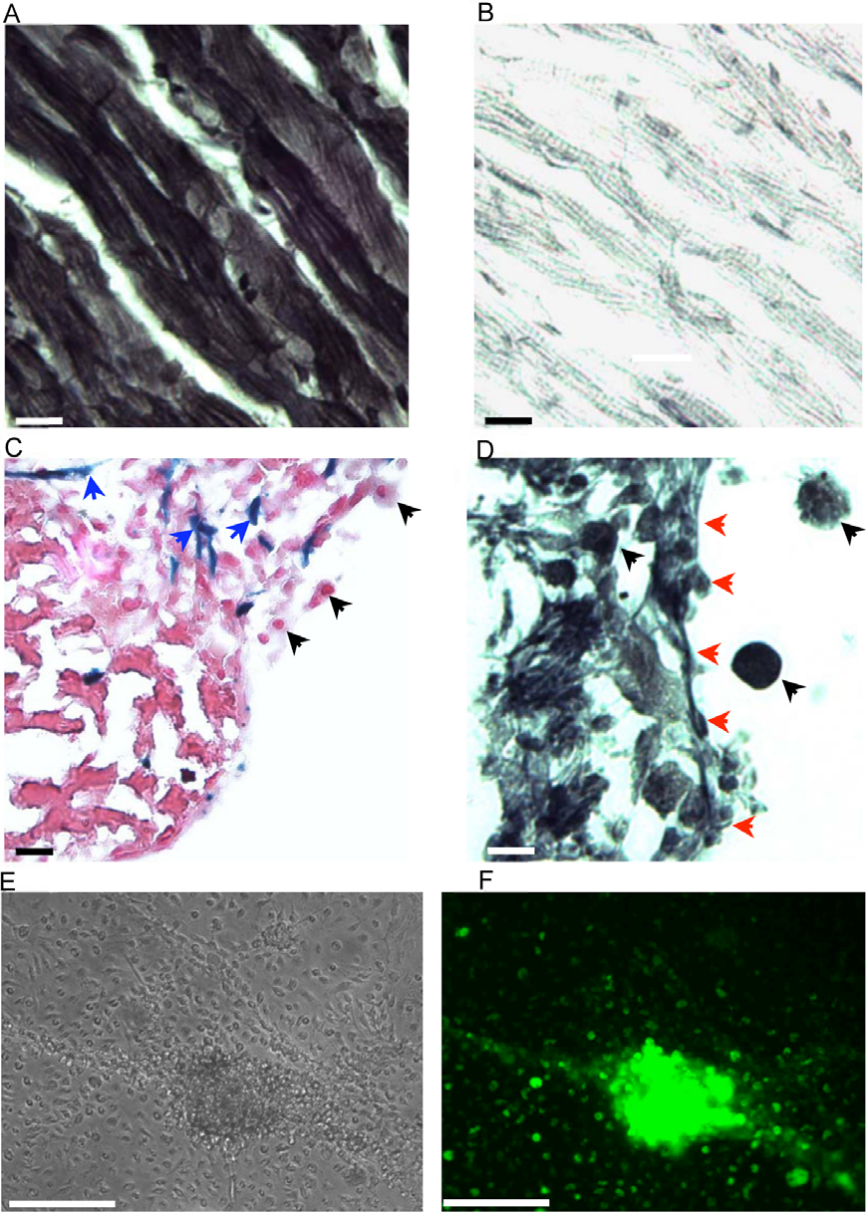
Cardiac Explants and cells from MLC2v-Cre/ZEG double transgenic mice. (a) Demonstration of GFP immune reactivity in fresh uncultured heart tissue from MLC2v-Cre/ZEG hearts. Signal was developed with a nickel-enhanced DAB-reaction (blue/black; scale bar, 10 microns). (b) Minus anti-body negative control for the image shown in panel a. (c) Cultured cardiac explants from MLC2v-Cre/ZEG mice were stained simultaneously with the chromogenic dye X-gal (generates a blue signal in the presence of beta-galactosidase) and with nuclear fast red counterstain. The spindle-shaped fibroblast-like cells were beta-galactosidase positive (blue arrows) whilst the EDCs were beta-galactosidase negative (scale bar, 20 microns) (d) GFP immune reactivity in EDCs (black arrows). Signal was developed with a nickel-enhanced DAB reaction. Red arrows indicate the edge of the explant (scale bar, 5 microns). (e–f) Phase contrast and fluorescent illumination of the same field of cultured EDCs (scale bar, 100 microns).

A binary, conditional, cardiac restricted transgenic reporter system, the double heterozygous MLC2v-Cre/ZEG reporter mouse, was employed for cardiac specific lineage tracing. In the MLC2v-Cre mouse, the promoter of the ventricular isoform of myosin light chain drives the expression of cre-recombinase exclusively in ventricular cardiac myocytes[4]. Cre-recombinase recognises and excises lox-p sites in DNA. In the ZEG reporter mouse, the ZEG transgene results in the expression of ß-galactosidase (LAC Z) by most tissues via a ß-geo insert[5], which is flanked by lox-p sites. Hence in the MLC2v-Cre/ZEG reporter mouse, the presence of cre-recombinase results in the excision of the ß-geo, activating the constitutional expression of GFP in the ventricular myocytes. On the other hand, non-myocytes express LAC Z. In MLC2v-Cre/ZEG double-transgenic mice, the vast majority (94±0.5%) of ventricular cardiomyocytes expressed eGFP (n = 3) due to the cardiomyocyte-restricted Cre recombinase as evidenced by the presence of anti-GFP immune reactivity (Figure 5a–b).

Cardiac explants from the MLC2v-Cre/ZEG mice were cultured. Fibroblast-like cells and EDCs were produced in a similar temporal pattern as observed with the non-transgenic hearts above. The interstitial fibroblast-like cells in cultured explants expressed beta-galactosidase as evidenced by blue staining with the chromogenic substrate X-GAL (Fig. 5c, see blue arrows), whilst the budding EDCs did not (black arrows). Anti-GFP immune reactivity was detected in the budding EDCs (Figure 5d) using DAB staining to further confirm that the GFP epi-fluorescence detected in remote EDC colonies, but not in the underlying fibroblast outgrowths (Fig. 5e–f), was not due to autofluorescence.

**Figure 6:**
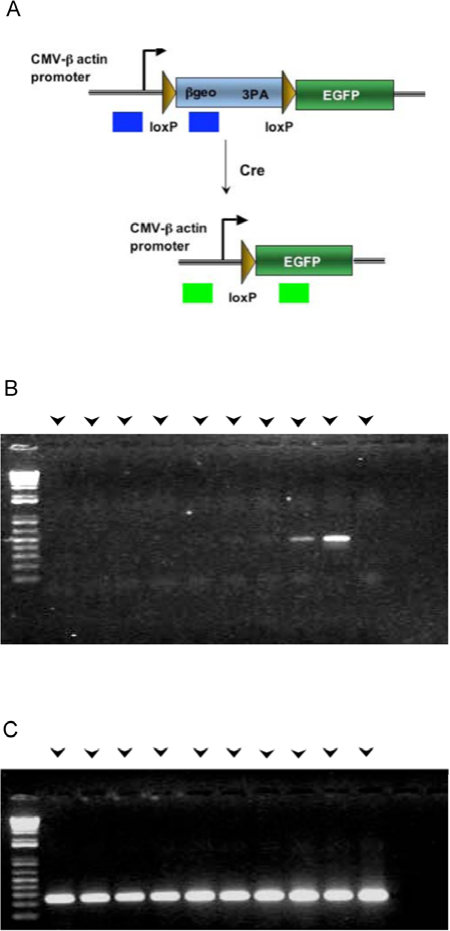
Analysis of DNA from MLC2v-Cre/ZEG double transgenic mice. (a) Structure of the ZEG reporter transgene before and after Cre recombinase-mediated deletion of the beta-galactosidase encoding sequences (adapted from gttf.uchc.edu/images/Cre%20marker/Slide1.jpg). Blue and black arrows indicate the positions of the PCR amplification primers used to distinguish native (450 bp amplification product) and recombined (450 bp amplification product) transgenes. (b) PCR amplification products using oligonucleotide primers to detect the Cre-mediated recombination event. DNA was from EDCs (lanes 1–4) and their corresponding explants (lanes 5–8), a positive control double transgenic mouse heart (lane 9) and a negative control double transgenic mouse kidney (lane 10). (c) PCR amplification products using oligonucleotide primers to detect the native ZEG reporter transgene. DNA samples were as in panel b.

Collectively, these results suggest that EDCs exhibit a limited number of cardiac attributes. Routine PCR control experiments were performed to confirm that the GFP immune reactivity and epifluorescence resulted from Cre-mediated activation of the ZEG reporter transgene (Figure 6a). Surprisingly, PCR primers designed to amplify a 450 bp fragment specific for the ZEG reporter following Cre-mediated recombination did not give rise to the expected amplification product when using DNA prepared from EDCs (Figure 6b, lanes 1–4), although the product was readily detected with DNA prepared from a freshly obtained MLC2v-Cre/ZEG double-transgenic mouse heart (Figure 6b, lane 9). The recombination-specific amplification product was only faintly detected in the explants (Fig. 6b lanes 5–8) due to selective cardiomyocyte death and ensuing nuclear loss (see Figure 1). In contrast, a 250 bp amplification product corresponding to the non-recombined ZEG allele (Figure 6a) was readily detected in all samples, including the EDCs, indicating that amplification-competent DNA was present (Figure 6c; strong signal was detected with explant DNA due to the survival on non-cardiomyocytes). These results unexpectedly showed that cre-mediated recombination of the ZEG transgene, and, concomitantly, activation of the eGFP reporter, had not occurred in the EDCs, in spite of the presence of GFP immune reactivity and epifluorescence.

**Figure 7:**
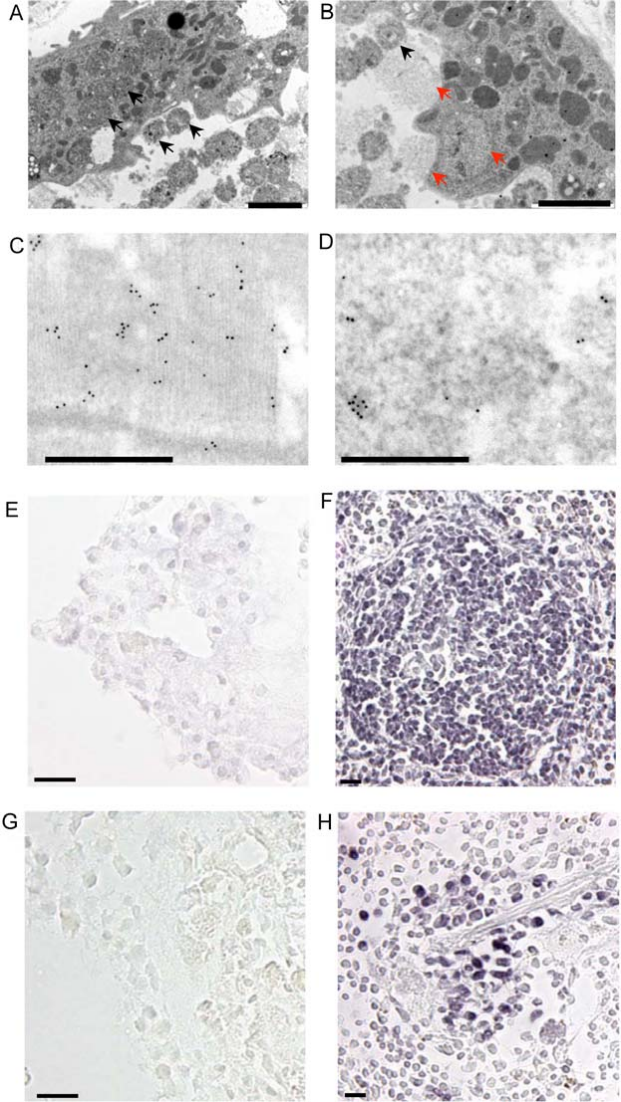
Additional ultrastructural and immune cytologic analysis of EDCs. (a–b) Apparent phagocytosis of cardiac myocyte mitochondria (a; black arrowheads) and sarcomeric structures (b; red arrowheads) by EDC-like cells within the interstitum of a cultured explant from an MLC2v-Cre/ZEG double transgenic mouse heart. (Scale bar, 2 microns). (c–d) Immunogold labelling of GFP in fresh non-cultured MLC2v-Cre/ZEG cardiac tissue (c), and in an endocytic compartment within an interstitial EDC-like cell (d) (Scale bars, 0.5 microns) (e) EDCs did not stain with an anti-CD 45 antibody (signal developed with a nickel-enhanced DAB reaction; scale bars, 20 microns) however (f) mouse spleen germinal centres processed in an identical manner as the EDCs in panel as expected showed a positive signal. (g) EDCs did not react with an anti-MAC-1 antibody (signal developed with a nickel-enhanced DAB reaction; scale bars, 20 microns) however (f) mouse splenic cords processed in an identical manner as the EDCs in panel g; had a positive signal.

Additional ultrastructural analyses were performed to ascertain the basis for GFP immune reactivity in EDCs from MLC2v-Cre/ZEG double-transgenic mice. Examination of EDCs within explant tissue at higher magnification revealed structures suggestive of active phagocytosis of mitochondria (Figure 7a, black arrows) and of sarcomeres (Figure 7b, red arrows). This view was supported by anti-GFP immunogold staining, which showed immune reactivity over what appeared to be an endocytic compartment within an EDC (Figure 7c–d). In light of these observations, the EDCs were tested for the presence of leukocyte and macrophage markers. EDCs did not express the pan-leukocyte marker CD45 or the macrophage marker MAC-1 (Figure 7e–h; sections of mouse spleen were used as the positive control).

### EDCs Do Not Differentiate Into Functional Cardiomyocytes in the Injured Heart

**Figure 8:**
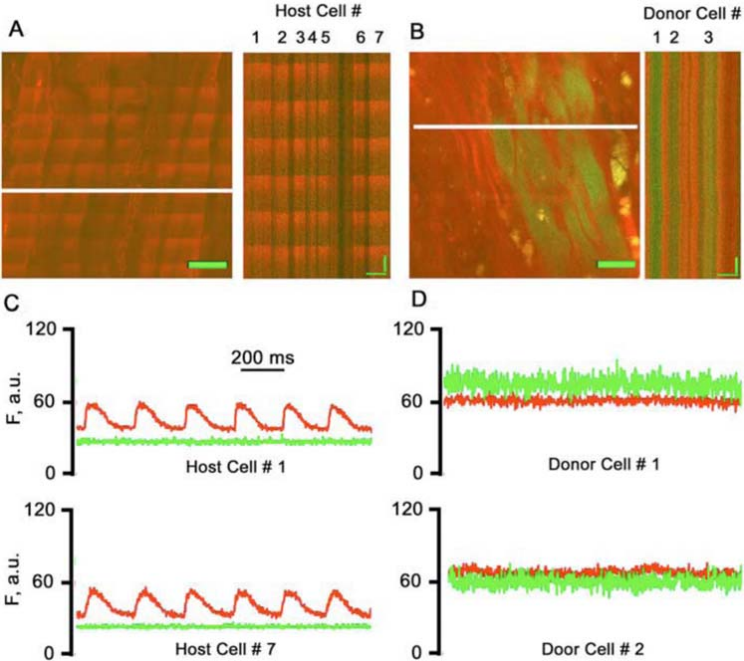
TPME analyse of hearts engrafted with EDCs from Actin-eGFP transgenic mice. (a) Full-frame TPME image (left panel) and line-scan image of myocardium in the infarct border zone of an engrafted heart. Host cardiomyocytes readily exhibit electrically evoked, synchronized calcium transients in a 1∶1 fashion during field stimulation at 4 Hz. Under these conditions, cardiomyocyte excitation is independent of cell-to-cell action potential propagation. Calcium transients appear as ripple-like wavefronts. Scale bar, 20 microns. (b) Electrical field stimulation of the same heart did not induce calcium transients in eGFP-expressing donor cell-derived cells or in non-expressing cells in an area contiguous to that shown in panel A, suggesting absence of functional cardiomyocytes in this field of view. Note the two horizontal red lines in the left panel which correspond to localized photodamage that had occurred during preceding line scans and are not to be confused with action potential-induced calcium transients. (c–d) Time course of the red (rhod-2) and green (eGFP) fluorescence in host cardiomyocytes (c) and donor-derived cells (d) obtained by spatially averaging the respective line-scans in panels a and b, respectively. No action potential-induced calcium transients were recorded in eGFP-expressing cells, whereas host cardiomyocytes show synchronized transients with identical kinetics.

Although the data presented above indicate that EDCs do not exhibit substantive spontaneous cardiomyogenic activity *in vitro*, it remains possible that they might exhibit such activity in the setting of myocardial injury *in vivo*. To directly address this, EDCs were prepared from cardiac explant cultures of actin-eGFP mice, and transplanted into the peri-infarct border of syngeneic non-transgenic mice following permanent ligation of the coronary artery. Three weeks later, the hearts were harvested, perfused with the calcium sensing dye rhod-2, and subjected to field stimulation for imaging with the Two Photon Molecular Excitation (TPME) Microscopy. Host cardiomyocytes (which exhibited red fluorescence due to the rhod-2 dye) were readily identified by their rod shaped morphology (Figure 8a, left panel). Data acquired in line-scan mode revealed transient increases in rhod-2 fluorescence, indicative of stimulation-evoked intracellular calcium transients (Figure 8a, right panel; the corresponding integrated traces are shown in Figure 8c). The transplanted cells were readily identified by green eGFP fluorescence (Figure 8b, left panel). As can be seen from the line scan data and the corresponding integrated traces (Figure 8b, right panel and Figure 8d, respectively), the eGFP-expressing donor cells lacked intracellular calcium transients, even under field stimulation (n = 3 transplanted hearts, more than 50 individual donor cells were analyzed). These data indicate that, although the donor cells survived for at least 3 weeks following transplantation into injured hearts, they lack structural correlates of myocyte-specific excitation-contraction machinery.

## Discussion

The EDCs described here share several characteristics of cardiac explant derived progenitors described by other investigators[1]–[3]. In agreement with previous works, EDCs are a distinct population of small, round highly refractile cells which appeared 1–3 weeks after cardiac explants were cultured despite some differences in culture conditions. The appearance of EDCs was also preceded by the formation of a layer of fibroblast-like smooth muscle actin positive cells. Transmission EM analyses revealed the presence of interstitial cells within the explant with ultrastructural attributes very similar to the EDCs, suggesting that these cells track through the interstitium towards surface layer, and then emerge from the explant. Preliminary expression studies detected GATA-4 mRNA (a cardiomyogenic transcription factor) and vimentin and sarcomeric actinin protein, consistent with the cardiomyogenic activity reported for the other explant-derived cells[1], [2].

Given these promising attributes, it was rather disappointing that analyses with the MLC2v-Cre/ZEG reporter system failed to support the presence of cardiomyogenic activity in cultured EDCs (as evidenced by the absence of cre-mediated recombination of the reporter locus). To eliminate confounding effects of autofluorescence, DAB immunostaining was performed with adequate negative controls. There was no background staining where the primary antibody was omitted. Backgound endogenous peroxidase activity was also excluded by peroxidase pre-treatment. The presence of GFP as well as vimentin and sarcomeric actinin immune reactivity, appeared to result from phagocytotic activity. This view was supported by ultrastructural analyses which demonstrated the presence of mitochondria, cardiac myocyte sarcomeric structures, and anti-GFP immunogold staining within the endocytic vesicles of the EDCs. Although the EDCs appeared to exhibit phagocytic activity, they did not express leukocyte or macrophage markers. It is also possible that the observed GFP fluorescence resulted from nanotube formation between EDCs and cardiomyocytes, as was recently reported for cardiomyocyte/endothelial progenitor cell co-cultures[6], although it is unlikely that molecules transported in this manner would localize to endocytic vesicles.

The notion that EDCs are macrophage-like cells is also supported by the observation that explants generated from perfused hearts gave rise to fibroblast-like out-growths but not to EDCs. Thus, the EDCs are more likely to arise from circulating cells which migrated into the heart, rather than from a static component of the cardiac interstitium. A recent study has suggested a similar origin for c-kit+ cardiac progenitors[7]. This idea was further supported by another study, suggesting that hematopoietic progenitor cells trafficked through blood and other peripheral tissues as part of immunosurveillance[8]. The main difference between EDCs & cells described in the latter study[8] is that EDCs did not express c-kit, Sca-1 nor CD45. Although it is possible that the loss of EDCs from perfused heart explants could be due to the wash-out of a requisite growth factor, this is unlikely given the high serum content employed in the culture media.

The absence of electrically-evoked calcium transients in EDCs following transplantation into infarcted hearts provided additional evidence for the lack of cardiomyogenic potential. Since the cell culture techniques used to generate the EDCs was similar to those employed in earlier studies, which obtained positive results[1], [2], it is unlikely that *in vitro* culturing *per se* resulted in a loss of cardiomyogenic potential. It is however possible that culturing in suspension (i.e., generation of so-called cardiospheres) is required to unmask cardiomyogenic potential. Indeed, cardiomyogenesis proceeds much more efficiently when ES cells are differentiated in suspension culture rather than in monolayer culture. Regardless of such theoretical activities, the inability of EDCs to form functional myocardium following transplantation into injured hearts strongly argues that their normal activity *in vivo* does not entail direct cardiomyogenic activity.

The initial observation of GATA-4 transcripts in EDCs from non-transgenic mice and GFP fluorescence in EDCs from MLC2v-Cre/ZEG reporter mice underscores potential difficulties encountered when examining cardiomyogenic differentiation *in vitro*. In the case of GATA-4 expression, it is noteworthy that previous analyses of mesenchymal derived cells showed partial activation of “myocyte specific” transcription in the absence of cardiomyogenic activity[9], [10]. Thus, transcriptional induction of a limited number of target genes is insufficient evidence of lineage induction. Although phagocytosis offers a ready explanation for the presence of GFP epifluorescence in the absence of reporter gene recombination in EDCs isolated from MLC2v-Cre/ZEG reporter mice, the absence of beta-galactosidase activity is problematic. This phenomenon of failed beta-galactosidase activity in the ZEG mouse lines has been previously described: beta-galactosidase activity was absent in the liver and lungs of ZEG lines, but robust GFP fluorescence was observed following cre-mediated recombination[5]. The author of that study suggested that low or absent expression of β-galactosidase may be an occasional phenomenon related to the poor expression of prokaryotic genes in eukaryotes. A similar mechanism could be at play in our model.

In conclusion, the present study demonstrated that, under our conditions, EDCs are not cardiac progenitors and raises caution about using cells from this source in human clinical trials. This study has also shown that the use of a limited number of parameters may not be sufficient to correctly identify the source and fate of cardiomyogenic progenitor cells.

## Materials and Methods

The investigations described in this article conform with the *Guide for the Care and Use of Laboratory Animals* published by the US National Institutes of Health (NIH Publication No. 85-23, revised 1996).

### Transgenic Mice

MLC2v-Cre/ZEG double heterozygous transgenic mice were generated by crossing the MLC2v-Cre mouse (generously provided by Dr C.A. Pritchard) with ZEG reporter mouse (purchased from Jackson laboratories, UK). In addition, actin-eGFP (generously provided by Professor L.J. Field), which ubiquitously express eGFP under the regulation of the beta-actin promoter[11], was also used.

### Explant Cultures

Isolated adult mouse hearts were diced into small tissue pieces less than 1 mm^3^ in size. The explants were vigorously triturated with calcium free Hanks buffered saline solution. The explants were then resuspended and cultured in DMEM HAM /F12 supplemented with 10%FCS and LIF (cardiac explant culture medium). The media was changed every 4 days for up to five weeks.

### Mouse Heart Perfusion

Mice were anticoagulated with 200 units of heparin intraperitoneally. Fifteen minutes later animals were sacrificed by cervical dislocation, the heart dissected free and the aorta cannulated with the visual aid of a stereomicroscope. In an attempt to determine whether EDCs are cardiac or extra-cardiac, mouse hearts were perfused with a crystal buffered solution immediately after their excision, until no blood could be observed from the coronary sinus; the hearts were then diced into small muscle explants for culture for 3 weeks. The number of colonies of EDCs in a 6cm dish (one dish per heart) was counted and expressed a percentage of the total number of explants cultured (n = 4 hearts for each group). The results from perfused cardiac explants were compared to those of non-perfused cardiac explants cultured for the same period of time.

### BRDU Labelling and TUNEL Staining for Apoptosis

EDCs were grown on cover slips and fixed with 4% paraformaldehyde. Fixed cells were labelled with BrDU using a kit according to the manufactures instructions (Roche Diagnostics GmbH, Germany).

### Cytosine Arabinoside Treatment

Explants were cultured in explant medium containing 5 µM cytosine arabinoside (Sigma, UK), a cell cycle inhibitor for 2 weeks. The controls for these experiments were explants cultured in normal cardiac explant medium. After 2 weeks, the control explants were switched and cultured in media with the cytosine arabinoside, whilst the explants that had been cultured with cytosine arabinoside were crossed over to normal cardiac explant medium.

### Microscopy and Immunohistochemistry

Explants fixed in 2–4% paraformaldehyde, were embedded in paraffin and sectioned at 5 µm for H&E staining. EDCs were grown on cover slips and fixed with 4% paraformaldehyde for immunocytochemistry. Fixed explants were cryoprotected in 30% sucrose in PBS overnight, embedded in optimum cutting temperature (OCT) medium and sectioned at a thickness of 10 µm. Primary antibodies used included: smooth muscle actin, vimentin, von Willebrand factor, c-kit (all from Abcam, UK), Sca-1, CD45 (both from Pharmingen, UK), α-sarcomeric actinin (Sigma, UK), MAC 1 and NG2 (both from Chemicon, USA). Secondary staining was performed using appropriate horseradish peroxidase (Zymed Laboratories, USA), Vector Impress and DAB kits (Vector, UK) according to the manufacturer's instructions. Bright field images were acquired using the Leica DM5000b microscope and DSC 400C camera system (Leica Microsystem, UK), and fluorescent images were obtained using the Zeiss Axiovert 200M microscope (Zeiss, UK).

### X-gal Staining

Cryoprotected explants were embedded in OCT media and sectioned at 10 µm. For detection of LAC-Z, sections were immersed in X-GAL solution [comprising of 1 mg/ml 5-bromo-4-chloro-3-indolyl–d-galactoside (X-GAL), 5 mM potassium ferricyanide, 5 mM potassium ferrocyanide, and 2 mM magnesium chloride in PBS-all reagents from Sigma, UK], at 37°C overnight. LAC-Z stained cardiac explants from MLC2v-Cre/ZEG were counterstained with nuclear fast red (Vector, UK).

### Transmission EM

For Transmission EM (Simens 102 Transmission Electron Microscope, Simens, UK), EDCs were fixed in 2.5% glutaraldehyde in Dulbecco's PBS and post-fixed in 0.5% osmium tetroxide in PBS. After serial dehydration in graded alcohol, specimens were immersed in propylene, and embedded in Spurs resin. For immunogold staining tissue was fixed in 4% paraformaldehyde and embedded in LR White™ embedding resin before standard labelling with immunogold.

### DNA Extraction and PCR

Explants and their cells were trypsinised. The cells were separated from their corresponding explants with a 40 µm filter. DNA was extracted from the cells alone or from the explants alone using the DNeasy Kit (Qiagen, UK) according to instructions provided. GCTAACCATGTTCATGCCTTC and GTTTTCCCAGTCACGACGTT were the forward and reverse primers for the intact floxed ZEG transgene, with an expected product size of 250 bp. GCTAACCATGTTCATGCCTTC and GTAGGTCAGGGTGGTCACGA were the forward and reverse primers for the cre-deleted ZEG transgene with a product of 450 bp.

### RNA Isolation and RT-PCR

RNA was extracted using the Sensicript RNA Isolation Kit (Qiagen, UK) according to the manufacturer's instructions. For RNA extraction, EDCs were detached from the periphery of the explant at 3 weeks, when the EDCs are the predominant cell type produced from the explant by gentle agitation of the explants in 24 well culture plates. Only wells with EDCs were selected for RNA isolation based on physical appearance by visual inspection. Excess DNA was cleaned up from the preparation by use of RNase free DNase. Reverse transcription and the cDNA obtained stored at −20°C for PCR under the appropriate conditions. EDCs were tested for expression of GATA-4, MyoD, ANF and NKx2.5, using primers from Invitrogen, UK (See table S1). β-actin (Invitrogen, UK) was used as control[12].

### Myocardial Infarction

A left thoracotomy and permanent ligation of the left anterior descending artery was carried out in wild type mice as previously described[13]. Approximately 100 000 cultured cells suspended in 20 µl of PBS from the explants were injected into the border zone in a single injection. After 3 weeks the hearts were excised for immunohistochemistry or for TPME microscopy to assess coupling of the grafted cells as previously described[14]. For immunohistochemistry, the hearts were then fixed with 2–4% paraformaldehyde and stained as described above.

### TMPE Microscopy Imaging of Intracellular Calcium Transients in Intact Hearts

Preparation of the hearts carrying grafts of cardiospheres and TPME imaging was performed as described previously[15]. Images were recorded with an MRC 1024 laser scanning microscope (Bio-Rad Laboratories Inc., USA). Langendorff-perfused hearts were loaded with the fluorescent calcium indicator rhod-2 using the cell membrane permeable AM-ester of the dye (10 µmol/L rhod-2-AM for 10 minutes). Illumination for two-photon excitation was provided by a mode-locked Ti:sapphire laser (Spectra-Physics, USA); the excitation wavelength was 810 nm. Hearts were imaged through a Nikon x60 1.2 numerical aperture water-immersion lens with a working distance of 200 µm. Emitted light was collected by two photo-multiplier tubes fitted with narrow bandwidth filters for 560–650 nm and 500–550 nm, respectively. Images were collected at a resolution of 0.43 µm/pixel along the *x* and *y* axes. For full-frame mode analyses (512×512 pixels), hearts were scanned at 1.46 and 0.73 frames per second on horizontal (*x*, *y*) planes, and the resulting images were digitized at 8-bit resolution and stored directly on the hard disk. For line-scan mode analyses, hearts were scanned repetitively at 500 Hz along a line spanning at least two juxtaposed cardiomyocytes. Line-scan images were then constructed by stacking all lines vertically. Post-acquisition analysis was performed using MetaMorph software version 4.6 (Universal Imaging Corp, USA).

During TPME imaging, hearts were perfused with oxygenated normal Tyrode's solution containing 50 µmol/l cytochalasin D to eliminate contraction-induced movement. Records were made during electrical point stimulation or during electrical field stimulation as described previously[15], [16]. Plots of changes in [Ca^2+^]_i_ as a function of time were obtained by averaging rhod-2 fluorescent intensities along the scan lines in line-scan images.

## Supporting Information

Figure S1 (2.91 MB TIF)

Table S1 (0.02 MB DOC)
