# Supplementary figures and images for: Lineage Tracing of Cardiac Explant Derived Cells
Source: PLoS One. 2008 Apr 16;3(4):e1929. doi: 10.1371/journal.pone.0001929 (PMC2288675; doi:10.1371/journal.pone.0001929)

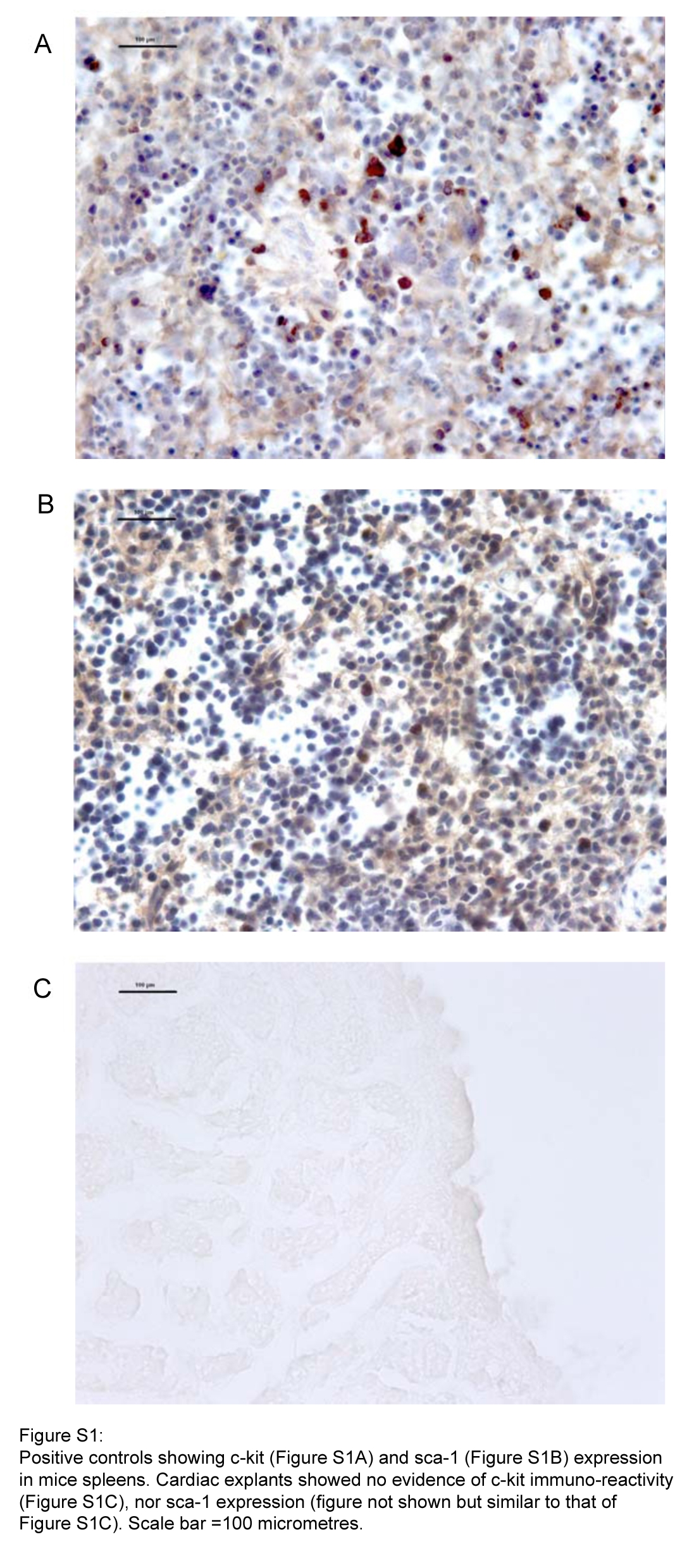

Supplement: Figure S1 — (2.91 MB TIF) [file pone.0001929.s001.tif]
