# Supplementary material for: Lineage Tracing of Cardiac Explant Derived Cells
Source: PLoS One. 2008 Apr 16;3(4):e1929. doi: 10.1371/journal.pone.0001929 (PMC2288675; doi:10.1371/journal.pone.0001929)
Supplement: Table S1 — (0.02 MB DOC) [file pone.0001929.s002.doc]

## Supplementary table 1: Primer Sequences and methods used for RT-PCR on EDCs

Primer Pairs Product Size

GATA4 5'-AAGACGCCAGCAGGTCCTGCTGGT-3' 340bp

5'-CGCGGTGATTATGTCCCCATGACT-3'

ANF 5'-TTGGCTTCCAGGCCATA-3' 282bp

5'-AAGAGGGCAGATCTATCGGA-3'

MyoD 5'-GGAAGAGTGCGGCTGTGT-3' 150bp

5'-CTGTTCTGTGTCGCTTAGGG-3'

Nkx2.5 5'-CAGTGGAGCTGGACAAAGCC-3' 217bp

5'-TAGCGACGGTTCTGGAACCA-3'

β-actin 5'-ATCGTGGGCCGCCCTAGGCACCA-3' 243bp

5'-TTGGCCTTAGGGTTCAGAGGGG-3'

For GATA-4 and MyoD, PCR were performed over 36 cycles, consisting of an initial denaturation at 94°C for 5 minutes, then 94°C for 30 seconds, 58°C (GATA-4) or 60°C (MyoD) for 30 seconds, 72°C for 30 seconds, and was terminated by a final extension at 72°C for 8 minutes. For ANF, the sample was initially denatured at 94°C for 4 minutes, followed by 5 cycles of denaturation at 94°C for 45 seconds, annealing at 57°C, 58°C, and 60°C, for 1 minute each, extension at 72°C for 2 minutes, and 30 cycles of denaturation at 94°C for 45 seconds, annealing at 50°C for 1 minute, extension at 72°C for 2 minutes, and 72°C for 10 minutes. Nkx2.5 PCR consisted of denaturation at 94°C for 5 minutes followed by 30 cycles of 95°C for 30 seconds, 58°C for 1 minute 30 seconds, 72°C for 1 minute, and a final extension at 72°C for 7 minutes. β-actin was used as control. The PCR products were size fractionated by 1.8% agarose gel electrophoresis.
